# Oral behaviors in Chinese temporomandibular disorder patients: insights from exploratory and confirmatory factor analyses

**DOI:** 10.3389/fneur.2024.1522057

**Published:** 2024-12-18

**Authors:** Tiqian Liu, Adrian Ujin Yap, Yanyu Sun, Yunhao Zheng, Tianqi Wang, Shiya Zeng, Zhen Liu, Xin Xiong

**Affiliations:** ^1^West China School of Stomatology, Sichuan University, Chengdu, China; ^2^State Key Laboratory of Oral Diseases, National Centre for Stomatology & National Clinical Research Centre for Oral Diseases, West China Hospital of Stomatology, Chengdu, Sichuan University, Chengdu, China; ^3^Division of Dentistry, Ng Teng Fong General Hospital and Faculty of Dentistry, National University Health System, Singapore, Singapore; ^4^National Dental Research Institute Singapore, National Dental Centre Singapore, Duke-NUS Medical School, Singapore Health Services, Singapore, Singapore; ^5^Affiliated Hospital of North Sichuan Medical College, Nanchong, China

**Keywords:** factor structure, oral behaviors, temporomandibular disorders, exploratory factor analysis, confirmatory factor analyses

## Abstract

**Aim:**

This study aimed to evaluate the factor structure of the Oral Behaviors Checklist (OBC) in Chinese temporomandibular disorder (TMDs) patients and compare the outcomes with those of Western patients. Additionally, it examined the correlations between different OBC subscale scoring methods.

**Methods:**

A total of 869 patients completed a survey that included demographic information, the Symptom Questionnaire, and OBC. This was followed by a clinical examination and diagnosis based on the Diagnostic Criteria for TMDs (DC/TMDs). Exploratory factor analysis, along with confirmatory factor analysis, was applied to waking-state oral behaviors, revealing two key factors: Chinese non-functional (C-NFA) and functional (C-FA) oral activities. Items were contrasted with those of Italian TMDs patients (I-NFA and I-FA), and subscale scores were computed, compared, and correlated using Kruskal Wallis and Post-hoc and Spearman’s rank-order correlation (*α* = 0.05).

**Results:**

Variations in NFA and FA items were observed between Chinese and Italian TMDs patients. For both NFA scoring methods, significant differences were noted between pain-related and intra-articular TMDs. The C-NFA and I-NFA, as well as C-FA and I-FA, scoring methods yielded scores with strong correlations (r > 0.8).

**Conclusion:**

NFA and FA subscale items were determined for Chinese TMDs patients. Despite item discrepancies, C-NFA and C-FA scores were strongly correlated with I-NFA and I-FA scores, respectively. The OBC can be effectively simplified for use with Chinese TMDs patients. Developing and validating an East–West short-form version of the OBC should be prioritized, given the variations in oral behaviors across countries and cultures.

## 1 Introduction

Temporomandibular disorders (TMDs) represent the second most common musculoskeletal condition, following chronic low back pain, affecting approximately 34% of the general population (1–3). These disorders are associated with considerable pain and dysfunction within the masticatory system. Based on the Diagnostic Criteria for TMDs (DC/TMDs), TMDs can be categorized into three types: intra-articular (IT), pain-related (PT), and combined (CT) (1, 4). The features of TMDs include facial and preauricular pain, temporomandibular joint (TMJ) sounds, and episodes of both closed and open jaw joint locking (5–7). Furthermore, TMDs are frequently associated with primary headaches, with a high prevalence of comorbidity between the two conditions (8, 9).

Given the complex etiology of TMDs, the biopsychosocial model is essential for understanding TMDs. Supported by the Orofacial Pain Prospective and Risk Assessment (OPERA) studies, this approach also serves a key role in effectively managing TMDs (10, 11). The model proposes that TMDs emerge from the dynamic interaction of biological, psychological, social, and behavioral factors (12, 13).

Oral behaviors (OB) can occur during sleep or wakefulness, with waking-state oral activities being either functional, such as chewing and speaking, or non-functional (parafunctional), such as teeth grinding and jaw clenching (14, 15). Although these OBs are typically harmless, an increase in their frequency or intensity can exceed physiological tolerance and potentially cause adverse effects on the health of the stomatognathic system including tooth wear/fracture, restorative complications, and the development of TMDs (15, 16). However, the relationship between OBs and TMDs remains inconclusive. While most research indicates a higher prevalence of OBs (17) in individuals with TMDs compared to those without, some studies suggest the opposite (5, 18, 19).

The assessment of OBs can be subject-based, clinically-based, and/or instrumentally-based, with self-reported questionnaires being the most widely used subject-based method (17, 20). Among these, the Oral Behavior Checklist (OBC), which consists of two sleeping-state and nineteen waking-state items, is a well-established tool for identifying and quantifying ‘jaw overuse’ behaviors (1, 21–23). Although the OBC is an integral part of the DC/TMDs Axis II protocol (psychosocial and behavioral aspects), not all items in the OBC are pertinent or commonly observed in individuals with TMDs (14). Furthermore, in both clinical practice and research settings, patients and participants often lack the patience to meticulously complete all items of the OBC due to its length. Recently, Donnarumma et al. used exploratory factor analysis (EFA) to determine the factor structure of the OBC among Italian TMDs patients. Their analysis identified two distinct groups of OBs during wakefulness, specifically six non-functional (NFA) and six functional (FA) oral activities, resulting in a markedly streamlined OBC (19). Genetic factors, along with race, culture, and socio-environmental influences, can affect OBs during both sleep and wakefulness (24). Therefore, OBs prevalent in Western TMDs patients may differ from those of Eastern TMDs patients. Considering the limited research on OBs in Eastern TMDs samples, a similar methodology was applied to investigate the factor structure in Chinese TMDs patients. The aims of our study were: (1) to evaluate the factor structure of the OBC in Chinese TMDs patients, (2) to compare this factor structure with that of Western TMDs patients, and (3) to explore the correlations between different OBC subscales scoring methods. We hypothesize that the factor structure of the OBC in Chinese TMD patients is consistent with that observed in Italian TMD patients and that there is a significant correlation between the two OBC subscale scoring methods.

## 2 Methods

### 2.1 Study population

This study was carried out using data from questionnaires. Participants were recruited from consecutive patients seeking TMDs treatment at the West China Hospital of Stomatology, Sichuan University, from May 2022 to May 2024. This study has been ethically approved by the review board of the West China Hospital of Stomatology at Sichuan University and conducted in accordance with the Declaration of Helsinki, with the project identification number WCHSIRB-D-2022-212. A sample size of at least 300 participants was chosen for conducting exploratory (EFA) and confirmatory (CFA) factor analyses of the OBC, following the guidelines of Myers et al. (25). The inclusion criteria were: (1) age 18 years or older, and (2) at least one Axis I TMDs diagnosis according to the TMDs diagnostic criteria (DC/TMDs). The exclusion criteria were: (1) indeterminate diagnoses; (2) history of orofacial trauma and/or surgeries; (3) non-TMDs conditions; (4) cognitive impairments or illiteracy, and (5) incomplete questionnaires. Participants were provided with the study information and informed consent was duly obtained.

### 2.2 TMDs subgroups

TMDs diagnosis was determined using the DC/TMDs Axis I protocol, which includes a symptom questionnaire (SQ), physical examination, and, when applicable, supplementary diagnostic imaging. The short form version of Chinese in the DC-TMD Translations was retrieved from the following source: https://ubwp.buffalo.edu/rdc-tmdinternational/tmd-assessmentdiagnosis/dc-tmd-translations/. The physical examinations were conducted by three trained and calibrated specialists who were proficient in DC/TMDs procedures. Participants were subsequently categorized into IT, PT, and CT groups.

### 2.3 Assessment of OBs

The Chinese version of the OBC questionnaire, a self-reported instrument designed to identify and quantify the frequency of various obsessive behaviors, was sourced from www.rdc-tmdinternational.org.OBs over the past month were evaluated using the OBC, with items scored on a 5-point response scale, ranging from 0 points for “none of the time” to 4 points for “4–7 nights per week” or “all of the time.” The total OBC score (OBC-TS), representing the overall level of jaw overuse behavior, was obtained by summing the scores for all 21 items and categorized into three levels: normal (0 to 16 points), low (17 to 24 points), and high (25 to 84 points). Scores for waking-state and sleeping-state OBs were calculated by summing the nineteen items for wakefulness and the two items for sleep, respectively. As sleeping-state OBs only contained two items, they were excluded during the EFA. The Italian scoring method for the NFA subscale (I-NFA) involved summing the scores for items 3, 4, 5, 6, 7, and 11, while the Italian FA (I-FA) was based on the sum of items 12, 13, 17, 18, 19, and 20. The corresponding items from the Italian and Chinese NFA and FA subscales were eventually compared, and the resulting scores were correlated.

### 2.4 Statistical analysis

Statistical analyses were carried out using the Statistical Package for the Social Sciences (SPSS) software, version 27.0 (IBM Corporation, Armonk, NY, USA), with the significance level set at 0.05. EFA was conducted to reduce the dimensionality of the dataset and identify the underlying factors that influence the observed variables. The dataset was randomly divided, with two-thirds allocated to the EFA for exploration (n = 579) and one-third allocated to the CFA for validation (n = 290). The validity of the factor analysis models was assessed using two tests: the Kaiser-Meyer-Olkin (KMO) test and Bartlett’s test of sphericity. While the KMO test evaluates the level of multicollinearity, with values ideally exceeding 0.5, Bartlett’s test assesses the likelihood that the initial correlation matrix is an identity matrix, with a significant result (*p* < 0.05) indicating that the data is suitable for factor analysis. Given the ordinal nature of OBC responses, the polychoric covariance matrix and varimax rotation were applied to estimate the latent trait. Items with factor loadings under 0.5 were excluded to enhance the robustness of the analysis. Based on previous research, two factors were chosen (18).

To verify the factor structure, CFA was conducted, with specific criteria for model fit: a chi-square statistic to degrees of freedom ratio less than 3 (χ^2^/df < 3) with a *p*-value greater than 0.05, Root Mean Square Error of Approximation (RMSEA) below 0.10, Root Mean Square Residual (RMR) below 0.08, and Standardized Root Mean Square Residual (SRMR) below 0.08. These criteria collectively evaluated whether the model fitted the OBC data (26).

OBC data were presented as both means with standard deviations and medians with interquartile ranges (IQR). The Kolmogorov–Smirnov test indicated that the OBC data were not normally distributed. Therefore, the Kruskal-Wallis test, followed by post-hoc Mann–Whitney U tests, was used to examine the differences in OBC subscale scores among the various TMDs subgroups. Relationships between different subscale scoring methods of the OBs were assessed using Spearman’s rank-order correlation (27). The correlation coefficients (*r*) were classified into four categories: small (≥0.1), medium (≥0.3), large (≥0.5) (28).

## 3 Results

### 3.1 The sample of temporomandibular disorder patients

**Figure 1:**
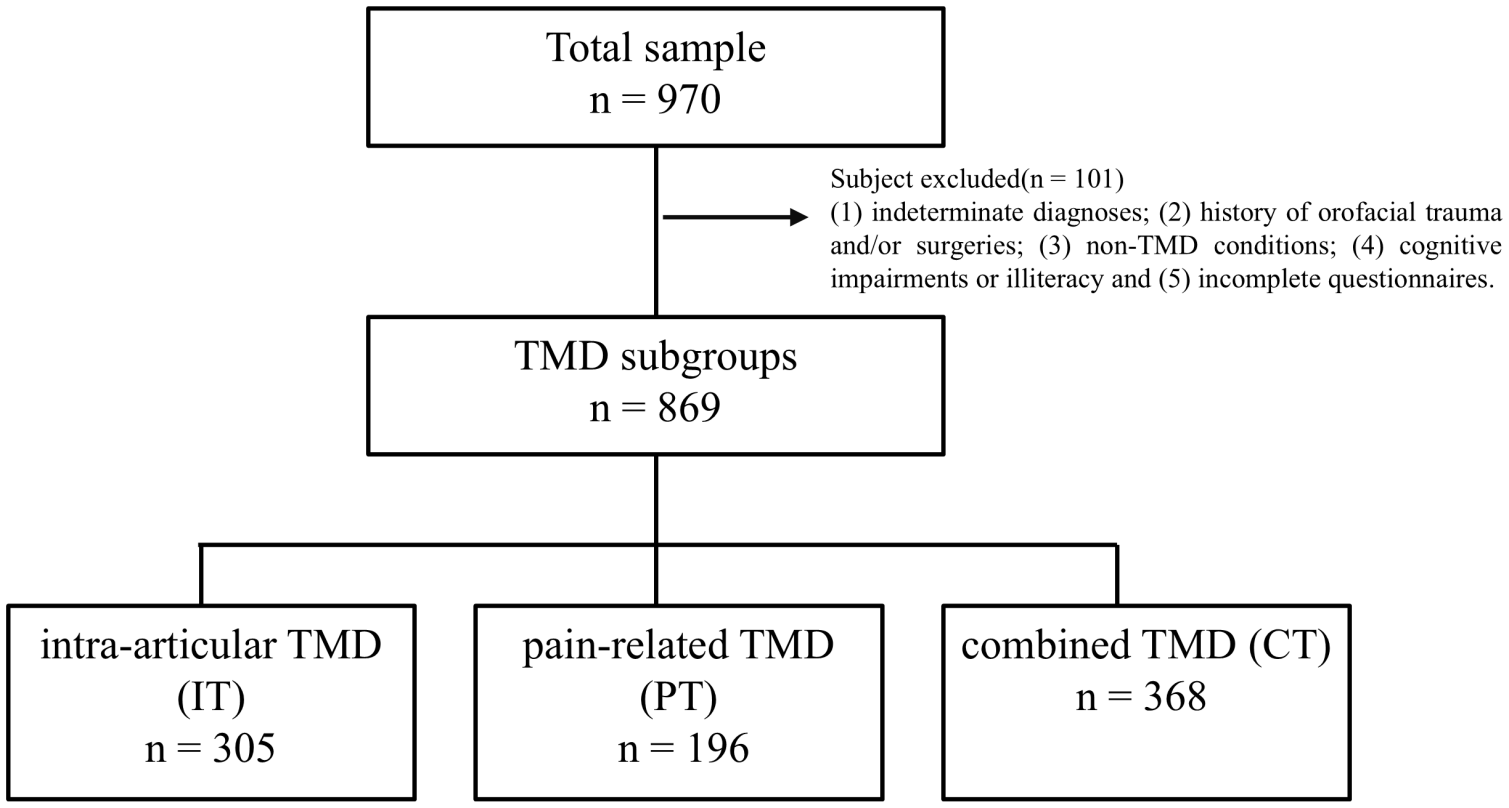
The sample of temporomandibular disorder divided three subgroups: intra-articular TMDs, pain-related TMD and combined TMD.

Out of 970 participants who initially met the inclusion criteria, 101 were omitted for meeting the specified exclusion criteria (Figure 1). The final sample of 869 participants, 78.83% of whom were female, had a mean age of 30.16 years (SD = 11). Among these, 35.1% (305) were diagnosed with IT, 22.6% (196) with PT, and 42.3% (368) with CT.

### 3.2 Exploratory factor analysis of OBC items in the waking state

The KMO value of 0.832 and the significant result from Bartlett’s test of sphericity (*p* < 0.001) confirmed that the correlation matrices were suitable for factor analysis and supported its use for the OBC dataset. Two distinct factors were identified in the EFA (Table 1). The first, termed Chinese-NFA (C-NFA), includes seven items (3, 5–10) representing non-functional oral behaviors like teeth grinding and holding actions. The second factor, termed C-FA, consists of five items (13, 15, 17, 18 and 21). Related to functional oral activities, such as chewing and singing.

**Table 1:** Exploratory factor analysis of oral behaviors checklists in the waking state.

Items	Description of items	Factor 1	Factor2
OBC_3	Grind teeth together during waking hours	0.512	
OBC_4	Clench teeth together during waking hours		
OBC_5	Press, touch or hold teeth together other than while eating (that is, contact between lower teeth)	0.543	
OBC_6	Hold, tighten or tense muscles without clenching or bringing teeth together	0.790	
OBC_7	Hold or jut jaw forward or to the side	0.676	
OBC_8	Press tongue forcibly against teeth	0.721	
OBC_9	Place tongue between teeth	0.544	
OBC_10	Bite, chew or play with your tongue, cheeks or lips	0.606	
OBC_11	Hold jaw in rigid or tense position, such as to brace or protect the jaw.		
OBC_12	Hold between the teeth or bite objects such as hair, pipe, pencil, pens, fingers and fingernails.		
OBC_13	Use chewing gum.		0.563
OBC_14	Play musical instrument that involves use of mouth or jaw (e.g., woodwind, brass, string instruments)		
OBC_15	Lean with your hand on the jaw, such as cupping or resting the chin in the hand		0.548
OBC_16	Chew food on one side only.		
OBC_17	Eating between meals (i.e., food that requires chewing)		0.679
OBC_18	Sustained talking (e.g., teaching, sales, and customer service).		
OBC_19	Singing		0.646
OBC_20	Yawning		
OBC_21	Hold telephone between your head and shoulders		0.580
Cumulative percentage		13.766%	32.696%

Exploratory factor analysis of Oral Behaviors Checklists in the waking state. OBC, oral behavior checklists. The polychronic correlation matrix and varimax rotation of exploratory factor analysis to explore the factor loading. Items with loading coefficients below 0.5 are ignored and not listed. The final rows represent cumulative percentages for Factor 1 and Factor 2 are reported. Oral behaviors during waking-state were selected for this analysis in OBC items.

### 3.3 The scatterplot of exploratory factor analysis loading of OBC items

**Figure 2:**
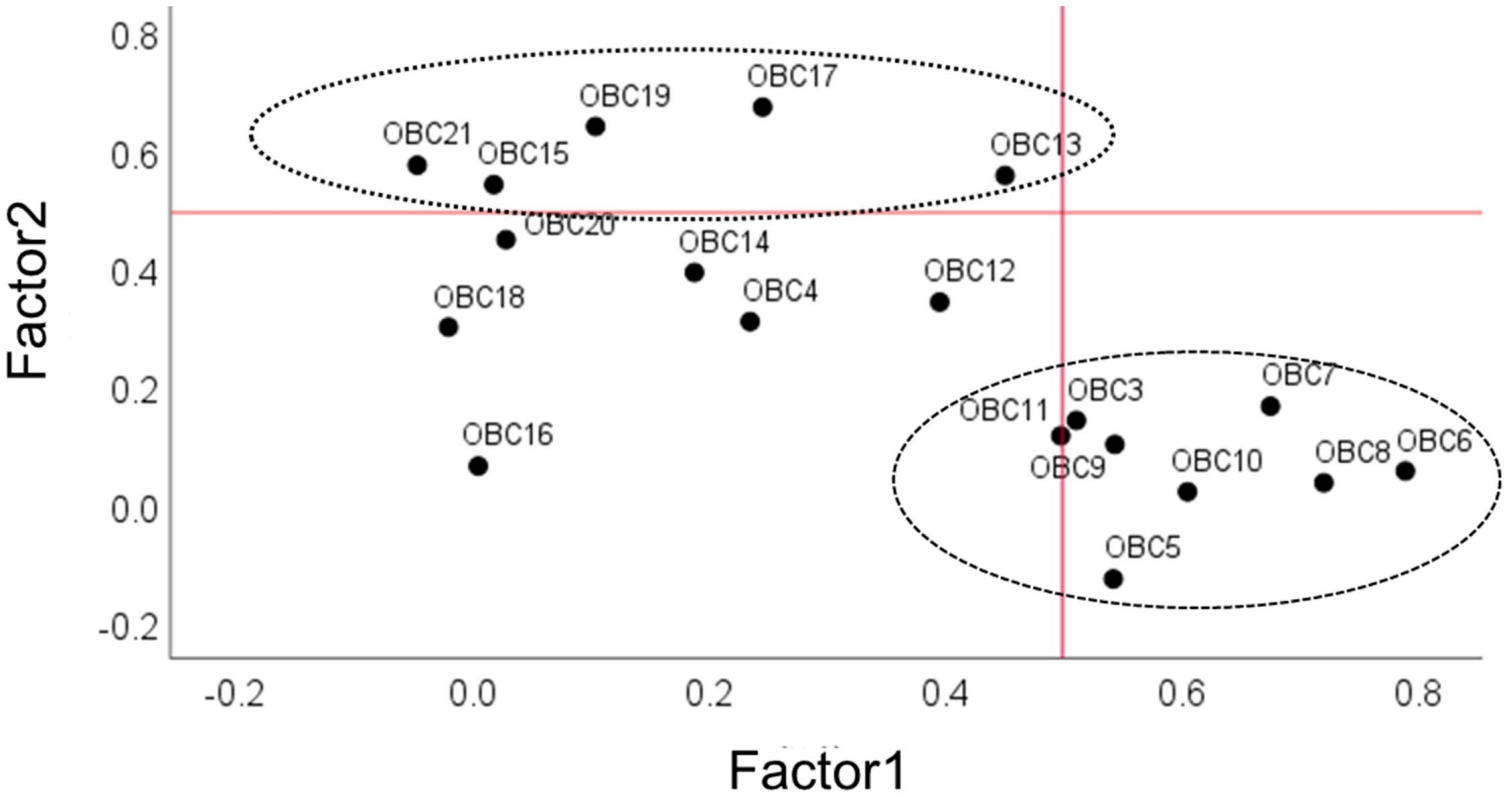
Scatterplot of exploratory factor analysis (EFA) loading of OBC items related to diurnal oral behaviors. On *x*-axis and *y*-axis, loadings are shown for factor 1 and factor 2. Numerals in the plot space refer to item numbers (see Table 2 for clarification). Circles with different line drawing represent OBC new scale (NFA and FA, see methods and results). Dotted line represents loading factor <0.5.

A scatterplot was used to visualize the associations between the OBC items and the two identified factors, with items 4, 11, 12, 14, 16, 18, and 20 excluded. These items were omitted because their factor loadings fell below the 0.5 threshold (Figure 2).

### 3.4 Confirmatory factor analysis of OBC items in TMDs patients

**Figure 3:**
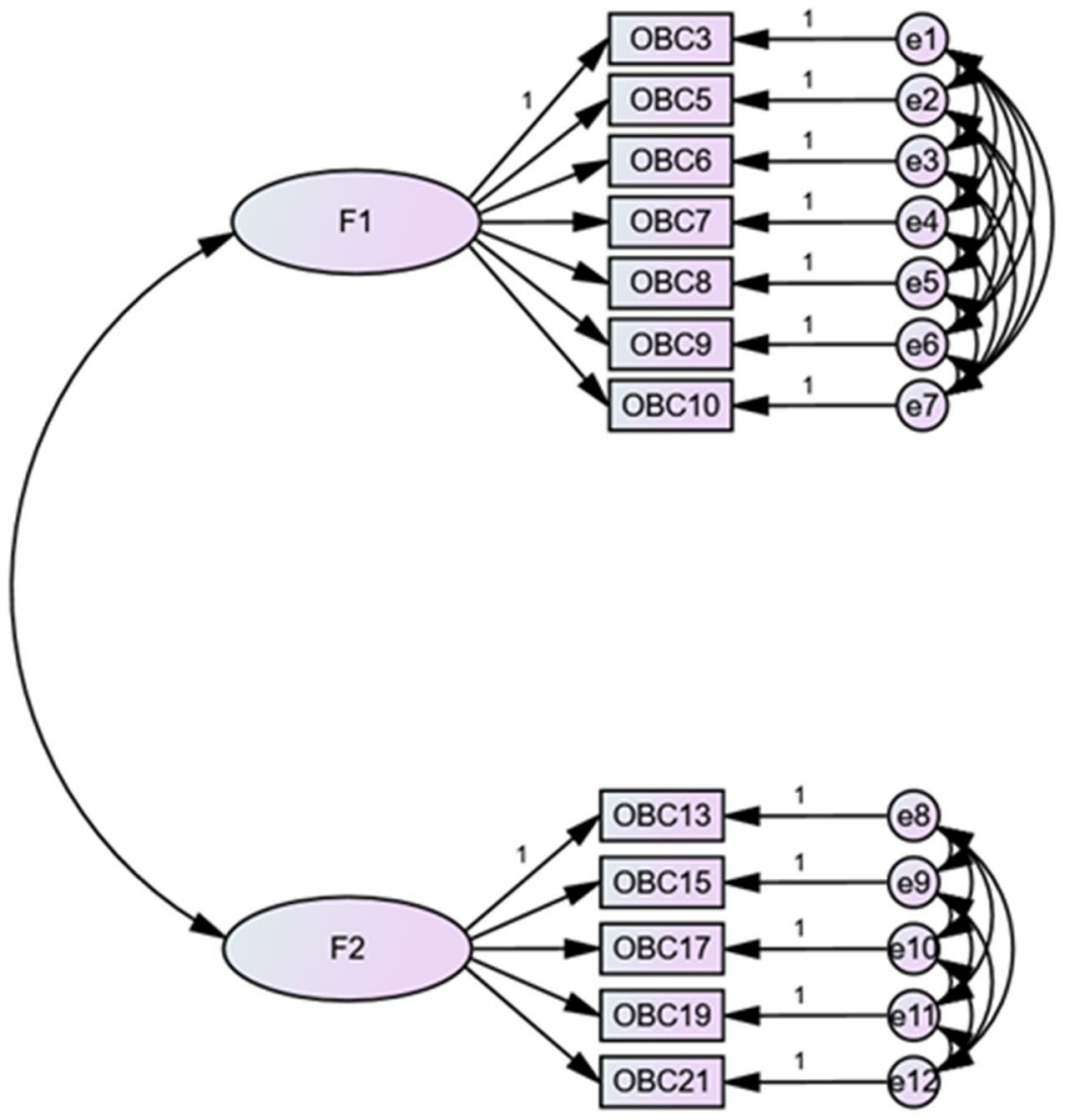
Model fit statistics for confirmatory factor analysis.

Figure 3 presents the CFA results with critical values indicating RMSEA <0.10, RMR <0.08, and SRMR <0.08, demonstrating a satisfactory goodness of fit.

### 3.5 The mean/median oral behaviors among three TMDs subgroups

Table 2 shows the statistically significant differences in C-NFA scores (PT > IT) and I-NFA scores (PT > IT) among the TMDs subgroups. However, no significant differences were observed in OBC-TS, waking-state OBs, sleeping-state OBs, C-FA, or I-FA scores across the three TMDs groups.

**Table 2:** Mean/median oral behavior among three TMDs subgroups.

Variable	All patients	IT	PT	CT	*p*-value Post-hoc
OBC-TS					
Mean (SD)	45.42 (9.09)	23.71 (9.47)	24.86 (8.71)	24.79 (8.95)	0.234
Median (IQR)	12	11	12	12	
Waking-state OB					
Mean (SD)	40.98 (8.67)	20.33 (9.06)	20.18 (8.28)	20.24 (8.53)	0.259
Median (IQR)	12.00	12.00	11.00	12.00	
Sleeping-state OB					
Mean (SD)	4.4 (1.13)	4.38 (1.06)	4.37 (1.07)	4.55 (1.22)	0.095
Median (IQR)	1.00	1.00	1.00	1.00	
C-NFA score					
Mean (SD)	14.01 (4.73)	13.42 (4.74)	14.87 (4.79)	14.05 (4.62)	0.035
Median (IQR)	7.00	6.00	7.00	7.00	PT > IT
C-FA score					
Mean (SD)	11.37 (3.00)	11.48 (3.05)	11.19 (2.91)	11.37 (3.01)	0.566
Median (IQR)	4.00	4.00	4.00	4.00	
I-NFA score					
Mean (SD)	18.13 (5.28)	17.48 (5.31)	18.88 (5.22)	18.29 (5.25)	0.011
Median (IQR)	8.00	11.00	7.00	7.00	PT > IT
I-FA score					
Mean (SD)	16.53 (3.82)	16.45 (4.74)	14.87 (4.79)	14.05 (4.62)	0.708
Median (IQR)	5.00	5.00	8.00	9.00	

IT, intra-articular TMDs; PT, pain-related TMDs; CT, combined TMDs; OBC, Oral Behavior Mean/median oral behavior among three TMDs subgroups. Checklists; SD, standard deviation; IQR, Inter quartile range; OB, oral behavior; C-NFA, Chinese no-functional activities; C-FA, Chinese functional activities; I-NFA, Italian no-functional activities; I-FA, Italian functional activities; Results of Kruskal Wallis and Post-hoc.

### 3.6 The correlations among the different subscale scoring methods for oral behaviors in TMDs patients

Table 3 displays the results of the correlation analysis, indicating a large relationship between OBC-TS and waking-state OBs (*r* = 0.99). Additionally, OBC-TS had large correlations with C-NFA (*r* = 0.80), C-FA (*r* = 0.75), I-NFA (*r* = 0.87), and I-FA (*r* = 0.84). Similarly, the waking-state OB score was strongly associated with C-NFA (*r* = 0.82), C-FA (*r* = 0.73), I-NFA (*r* = 0.88), and I-FA (*r* = 0.83). The large correlations were observed between C-NFA and I-NFA (*r* = 0.95) and between C-FA and I-FA (*r* = 0.82). The association between C-NFA and C-FA was small (*r* = 0.36), while the correlation between I-NFA and I-FA scores was medium (*r* = 0.51).

**Table 3:** The correlations among the different subscale scoring methods for oral behaviors in TMDs patients.

Variables	OBC-TS	Waking-state OB	Sleeping-state OB	C-NFA	C-FA	I-NFA	I-FA
OBC-TS	1						
Waking-state OB	0.99^***^	1					
Sleeping-state OB	0.42^***^	0.31^***^	1				
C-NFA	0.80^***^	0.82^***^	0.15^***^	1			
C-FA	0.75^***^	0.74^***^	0.35^***^	0.36^***^	1		
I-NFA	0.87^***^	0.88^***^	0.16^***^	0.95^***^	0.41^***^	1	
I-FA	0.84^***^	0.83^***^	0.36^***^	0.47^***^	0.82^***^	0.51^***^	1

OBC, Oral Behavior Checklists; TS, total score; OB, oral behaviors; C-NFA, Chinese no-functional activities; C-FA, Chinese functional activities; I-NFA, Italian no-functional activities; I-FA, Italian functional activities; Results of Spearman’s rank-order correlation. *Indicates *p* < 0.05, ** indicates *p* < 0.01, *** indicates *p* < 0.001.

## 4 Discussion

We applied EFA and CFA to investigate the factorial structure of OBC in Chinese TMDs patients. The primary findings of our study are as follows: (1) The waking-state component of the OBC comprised two key factors, C-NFA and C-FA. However, the specific items contributing to these factors differed from those reported in Italian patients. (2) Patients with PT showed significantly higher C-NFA and I-NFA subscale scores compared to those with IT. No statistically significant differences were found in OBC-TS, waking-state OB, sleeping-state OB, C-FA, or I-FA scores among the TMDs subgroups. (3) The scoring methods for C-NFA and I-NFA, as well as C-FA and I-FA, yielded scores with significant and strong correlations. In light of the above, the first research hypothesis was partially supported, while the second was fully endorsed. The relationship between OBs and TMDs remains a contentious issue in current research. While most studies indicate a higher prevalence of OBs among TMDs patients compared to non-TMDs patients, as well as in patients with PT, some studies have reported no significant differences between the two groups (18, 19, 29). Part of these discrepancies may stem from racial and cultural differences that influence the types of OBs and the presentation of TMDs (2). Therefore, the factor structure and items reported for Italian patients must be evaluated in their Chinese counterparts.

### 4.1 EFA and CFA

Although EFA, corroborated by CFA, showed that waking-state OBs could be divided into two key factors for both Chinese and Italian patients, C-NFA and C-FA comprised seven and five items, respectively, while both I-NFA and I-FA contained six items each. In addition to differences in the number of items, the specific OBs also displayed slight variation, reflecting ethnocultural disparities in oral activity patterns. For NFA, four items overlapped between Chinese and Italian TMDs patients (items 3, 5, 6, and 7), whereas for FA, only three items were shared (items 13, 17, and 19). These seven waking-state items, along with the two sleeping-state items, could potentially serve as a “universal” East–West short-form OBC.

Five items excluded in Chinese TMDs patients (items 4, 11, 12, 18, and 20) were present in their Italian counterparts. Of particular interest is item 4, “clenching teeth together during waking hours,” which had the high factor loading in the I-NFA (19). This may be because Chinese TMDs patients were avoiding teeth clenching as well as other behaviors that could trigger or intensify pain (29). Item 12, concerning the behavior of “holding between the teeth or biting objects such as hair, pipes, pencils, pens, fingers, and fingernails,” was more prevalent among children, despite the study’s primary focus on an adult population. Notably, the items “playing musical instruments involving the mouth or jaw” (item 14) and “chewing food on one side only” (item 16) were absent in both patient groups. The exclusion of item 14 may be due to most patients not playing woodwind, brass, or relevant string instruments, while a lack of awareness about unilateral chewing may partially account for the omission of item 16.

### 4.2 Comparison among TMDs subgroups

Although no significant differences were observed in OBC-TS, waking/sleeping state, and FA scores, Chinese patients with PT exhibited substantially greater NFA scores than those with IT, regardless of the scoring methods used. These findings align with studies indicating that NFA may increase the risk of developing PT (29, 30). However, they contrast with results from Korean TMDs patients, where no significant differences in NFA scores were observed using the Italian scoring method, suggesting possible ethnocultural variances even within East Asian TMDs samples (17). Among the NFAs, “holding teeth together during activities other than eating” was especially common in Chinese TMDs patients, reported in approximately one-third of cases (29). The previously esteemed “biopsychosocial” model of TMDs etiology emphasized the multifactorial contributions of genetics, environmental conditions, gonadal hormones, overall health status, jaw trauma, oral parafunctional activities, somatization, depression, and anxiety (31, 32). Given the methodological parallels, it is reasonable to suggest that a combination of genetic and cultural influences might have played a role in the observed differences in the factor structure of OBC between the Chinese and Italian TMDs patients.

### 4.3 Correlations between Chinese and Italian scoring methods

The strong correlation between OBC-TS and waking-state OBs was expected, as they encompassed 90% of all items. Likewise, associations between OBC-TS and waking-state OB scores with C-NFA, C-FA, I-NFA, and I-FA scores were also anticipated. The large correlations between C-NFA and I-NFA, as well as between C-FA and I-FA, can be attributed to the considerable overlap in their items, specifically four items for NFA and three for FA. Given these findings, East Asian studies that have applied the Italian scoring method for NFA and FA can be regarded as valid (17). Notwithstanding, the 9-item East–West short-form OBC warrants further exploration and testing of its psychometric properties in both clinical and community settings. While the correlation between C-NFA and C-FA scores was small, that between I-NFA and I-FA scores was medium. The contrast can be attributed to the different items used in the two scoring methods. According to the results of the EFA, the two factors are separate and thus generally not expected to be related.

### 4.4 Study limitations

Despite its strengths, including a large sample size, the use of the DC/TMDs, and a robust statistical methodology, the study had several limitations. First, the study involved only Chinese TMDs patients, which may reduce the applicability of its findings to other racial groups. Replicating the study in more diverse global TMDs populations is necessary to confirm its generalizability. Moreover, the study should be extended to include community samples to capture a broader representation of oral behaviors in individuals both with and without TMDs. Second, the OBC relied on self-reporting, which can be subject to recall, social desirability, and other forms of information bias. The results could also be influenced by individual perception and reporting accuracy. However, the large sample size may help offset this effect. Lastly, the analysis does not adjust for potential confounders such as age, gender, and socioeconomic status, which can affect oral behaviors and TMDs.

## 5 Conclusion

In summary, NFA and FA subscale items of the OBC were determined for Chinese TMDs patients using EFA and CFA. While the C-NFA comprised seven items (3, 5–10), the C-FA contained five items (13, 15, 17, 18 and 21). In spite of item discrepancies, C-NFA and C-FA scores were strongly correlated with I-NFA and I-FA scores, respectively. The OBC can be effectively simplified for use with Chinese TMDs patients. Developing and validating a “universal” East–West short-form version of the OBC should be prioritized, given the variations in oral behaviors across countries and cultures. Additionally, the “universal” short-form OBC needs to be verified in community samples. This approach would significantly enhance its applicability and relevance across diverse populations, fostering a deeper understanding and more effective assessment of oral behaviors and TMDs globally.

## Data Availability

The raw data supporting the conclusions of this article will be made available by the authors, without undue reservation.
